# High-Intensity Interval Training Snacks Improve Experimental Autoimmune Encephalomyelitis

**DOI:** 10.3390/ijms27156854

**Published:** 2026-07-30

**Authors:** Oswaldo C. Junior, Jully C. S. Viana, Lorranna E. A. Teixeira, Luiz G. V. Filho, Maria E. H. Corrêa, Mariana F. Andrade, Ana C. F. Sperandio, Giovanna P. Caetano, Philipe L. Brito, Maiara R. Salvador, Liliane S. Pinheiro, Marco Fabrício Dias-Peixoto, Alessandra P. Carli, Caio César de Souza Alves, Sandra B. R. Castro

**Affiliations:** 1 Faculdade de Medicina, Universidade Federal dos Vales do Jequitinhonha e Mucuri, Teófilo Otoni 39803-371, MG, Brazil; oswaldo.junior@ufvjm.edu.br (O.C.J.); jully.chayra@ufvjm.edu.br (J.C.S.V.); lorranna.evellyn@ufvjm.edu.br (L.E.A.T.); luiz.vilela@ufvjm.edu.br (L.G.V.F.); maria.hott@ufvjm.edu.br (M.E.H.C.); mariana.ferraz@ufvjm.edu.br (M.F.A.); ana.sperandio@ufvjm.edu.br (A.C.F.S.); giovanna.patricio@ufvjm.edu.br (G.P.C.); philipe.brito@ufvjm.edu.br (P.L.B.); liliane.pinheiro@ufvjm.edu.br (L.S.P.); caio.alves@ufvjm.edu.br (C.C.d.S.A.); 2 Instituto de Ciências da Vida, Universidade Federal de Juiz de Fora—Campus Avançado Governador Valadares, Governador Valadares 35032-620, MG, Brazil; maiara.info@gmail.com; 3 Departamento de Educação Física, Universidade Federal dos Vales do Jequitinhonha e Mucuri, Diamantina 39100-000, MG, Brazil; marcofabri@ufvjm.edu.br; 4 Instituto de Ciência Engenharia e Tecnologia, Universidade Federal dos Vales do Jequitinhonha e Mucuri, Teófilo Otoni 39803-371, MG, Brazil; alessandra.pcarli@ufvjm.edu.br

**Keywords:** multiple sclerosis, 3-HIIT, and immunomodulation

## Abstract

Several studies have shown that physical activity is associated with increased life expectancy and the prevention of chronic and neurodegenerative diseases. This study aimed to evaluate the impact of high-intensity interval training (HIIT) on the progression of an experimental model of multiple sclerosis. For this purpose, the murine model of experimental encephalomyelitis (EAE) was used in female C57BL/6 mice, divided into four groups: negative control (CN), positive control (EAE), HIIT once a day (1-HIIT), and HIIT snacks, three times a day (3-HIIT). The clinical score results demonstrated a significant reduction in the clinical score in mice subjected to 3-HIIT, confirmed by analyses of pro- and anti-inflammatory cytokines and histological analyses. These results provided solid evidence for the potential of HIIT protocols, especially 3-HIIT, to reprogram an autoimmune response in the CNS in mice with the EAE model, favoring a neuroprotective and immunoregulatory environment—evidence that may drive clinical research.

## 1. Introduction

Multiple sclerosis (MS) is a chronic inflammatory disease of the central nervous system (CNS), characterized by multifocal demyelination, axonal loss, and varying degrees of neurodegeneration, which lead to neurological deficits and disability over time. MS remains one of the leading causes of neurological disability in young adults, with increasing prevalence and incidence in various regions of the world, associated with genetic and environmental factors (vitamin D, smoking, viral infections such as Epstein–Barr) and demographic changes [1].

The pathophysiology of MS is complex and heterogeneous; models and human studies suggest an interaction between genetic predisposition and environmental triggers that leads to activation of innate and adaptive immune responses, resulting in myelin sheath destruction and axonal damage. Recent evidence points to the role of B cells and meningeal inflammation as central drivers of cortical pathology and clinical progression, especially in secondary-progressive forms, implying local immune responses that sustain neurodegeneration independent of classic inflammatory activity in the myelin [1].

Over the past two decades, there has been a significant expansion in pharmacotherapy for relapsing-remitting MS: oral medications (fingolimod, siponimod, cladribine, dimethyl fumarate) and monoclonal antibodies (natalizumab, alemtuzumab, anti-CD20 agents such as ocrelizumab, ofatumumab, and rituximab), each with distinct efficacy profiles [2,3]. Preventing MS before the onset of demyelination is a feasible goal, although difficult to achieve with current diagnostic resources. Furthermore, there is a lack of preventive interventions with adequate evidence of efficacy [4]. For preclinical studies of MS, the experimental autoimmune encephalomyelitis (EAE) model has been widely used [5,6,7,8]. It is characterized by an actively induced autoimmune response against myelin sheath proteins of the central nervous system (CNS) axons, such as myelin oligodendrocyte glycoprotein (MOG), which mimics some features of MS, allowing the study of new therapeutic strategies [9,10,11,12,13].

Given the general clinical picture of impairments expected in patients with MS, adjunctive therapies that can improve the patient’s quality of life and delay disease progression are important and are therefore the constant focus of studies. In this context, physical activity appears to have favorable effects on individuals with MS [1].

According to the ref. [14], adults should engage in approximately 150–300 min of physical activity per week to prevent the onset or progression of chronic diseases. In addition, High-Intensity Interval Training (HIIT) has been shown to improve cardiorespiratory fitness, glucose control, blood pressure, and cardiac function compared to more traditional forms of aerobic exercise [15]. The 1-HIIT and HIIT snacks (3-HIIT) study models refer to patterns of exposure with the same total training time, performed once a day (1-HIIT) or three times a day (3-HIIT), in shorter periods that add up to the same cumulative time. Studies in mice reinforce the notion that 3-HIIT is superior at improving cardiovascular function [16]. Furthermore, HIIT is a safe and potentially effective strategy for improving functional and cardiorespiratory fitness in patients with multiple sclerosis, especially those with lower disability [17].

In the present study, the results demonstrated that HIIT snacks appear to reprogram the autoimmune response in the CNS of mice induced in the EAE model, promoting a neuroprotective and immunoregulatory environment.

## 2. Results

### 2.1. Clinical Score of EAE

**Figure 1:**
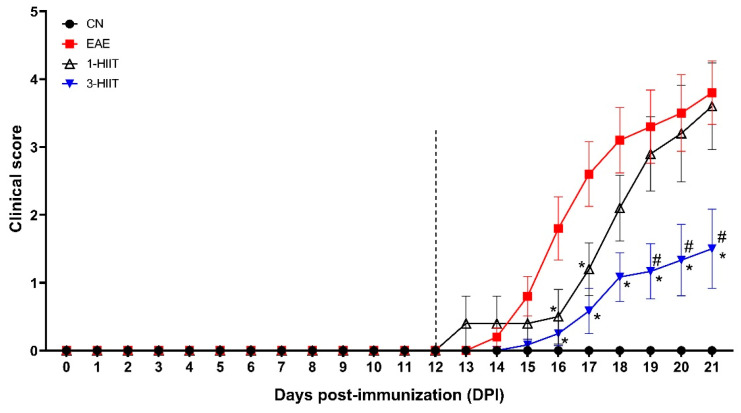
Clinical scores of animals induced with Experimental Autoimmune Encephalomyelitis (EAE). CN: Non-induced, non-exercised negative control; EAE: group induced with EAE and not exercised; 1-HIIT: group induced with EAE and exercised using the 1-HIIT protocol; 3-HIIT: group induced with EAE and exercised using the 3-HIIT protocol. Number of animals per group: *n* = 12. * *p* < 0.05 when compared to the EAE group. # *p* < 0.05 when compared to the 1-HIIT group. Data are expressed as mean ± standard error of the mean (SEM). Dashed line: onset of clinical symptoms. Significance assessed by two-way ANOVA with Tukey’s multiple comparisons test.

The 3-HIIT group showed a significant reduction in the clinical score compared with the other groups, with a maximum mean clinical score of 1.5 ± 0.584 on day 21 post-induction (Figure 1). As observed in Figure 1, the CN group showed no clinical signs of EAE throughout the entire evaluation period. Animals in the 1-HIIT group were the first to show clinical signs of EAE, starting on the 13th day post-induction (dpi), followed by the EAE group, with onset on 14 dpi, and, subsequently, the 3-HIIT group on 15 dpi. The 3-HIIT group had a lower mean clinical score than the EAE group starting at 16 dpi and remained statistically different until 21 dpi (*p* < 0.05). The 1-HIIT group exhibited fewer clinical signs than the EAE group on days 16 and 17 dpi (*p* < 0.05). Starting at 19 dpi, the 3-HIIT group continued to show a slower progression of clinical signs than the 1-HIIT group. No experimental data or endpoints were excluded from any of the groups.

### 2.2. Food Intake and Body Mass

**Figure 2:**
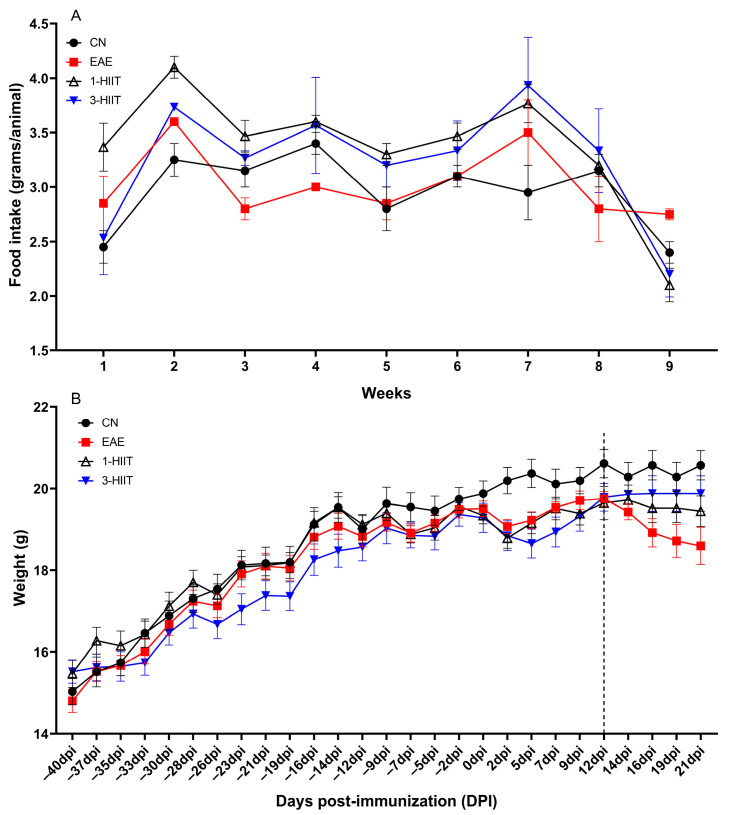
Food intake (**A**) and body weight (**B**) of C57BL/6 mice in the experimental groups induced with Experimental Autoimmune Encephalomyelitis (EAE). CN: Non-induced, non-exercised negative control; EAE: EAE-induced, non-exercised group; 1-HIIT: EAE-induced group exercised with the 1-HIIT protocol; 3-HIIT: EAE-induced group exercised with the 3-HIIT protocol. Number of animals per group: *n* = 12. Data are expressed as mean ± standard error of the mean (SEM). Dashed line: onset of clinical symptoms.

There were no differences in food intake (Figure 2A) or body weight (Figure 2B) between the groups.

### 2.3. Quantification of Cytokines in the Brain and Spinal Cord

**Figure 3:**
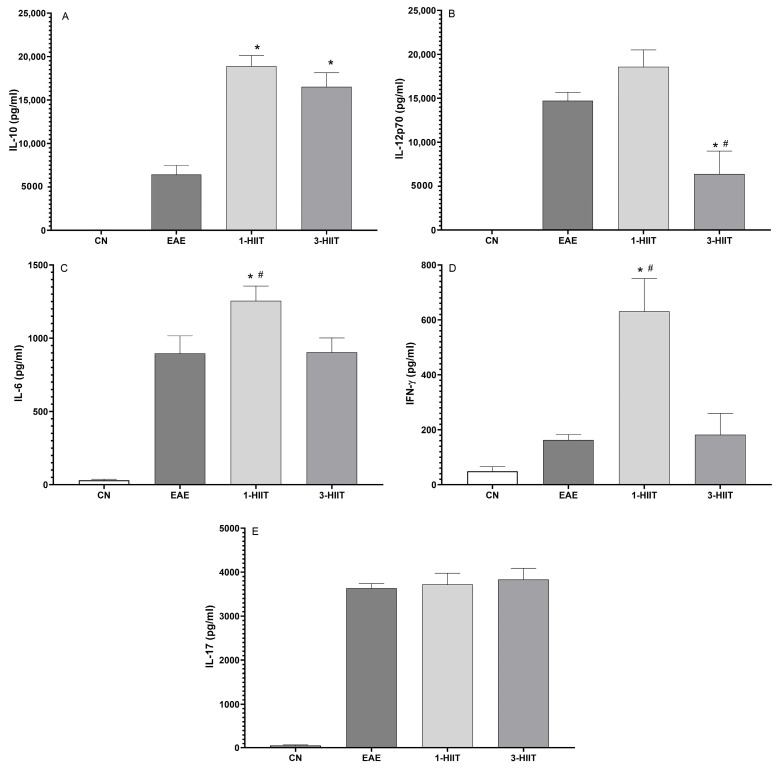
Cytokine levels in the brain homogenate supernatant of the experimental groups. (**A**) IL-10, (**B**) IL-12p70, (**C**) IL-6, (**D**) IFN-γ, and (**E**) IL-17. CN: Non-induced, non-exercised negative control; EAE: group induced with experimental autoimmune encephalomyelitis (EAE) and non-exercised; 1-HIIT: group induced with EAE and exercised using the 1-HIIT protocol; 3-HIIT: group induced with EAE and exercised using the 3-HIIT protocol. Number of animals per group: *n* = 8. * *p* < 0.05 versus EAE; # *p* < 0.05 1-HIIT versus 3-HIIT. Data are expressed as mean ± standard error of the mean (SEM). Significance assessed by one-way ANOVA with Fisher’s comparisons test or Kruskal–Wallis with Dunn’s comparisons test.

In the brain, the 1-HIIT and 3-HIIT groups showed a significant increase in IL-10 compared to the EAE group (Figure 3A). In addition, the 3-HIIT group showed reduced levels of IL-12p70 (Figure 3B). The IL-6 assessment showed that the EAE and 3-HIIT groups remained similar, whereas the 1-HIIT group had a higher concentration than both (Figure 3C). Regarding IFN-γ levels, the 1-HIIT group showed a significantly higher concentration than the others (Figure 3D). No change was observed in IL-17 production (Figure 3E).

**Figure 4:**
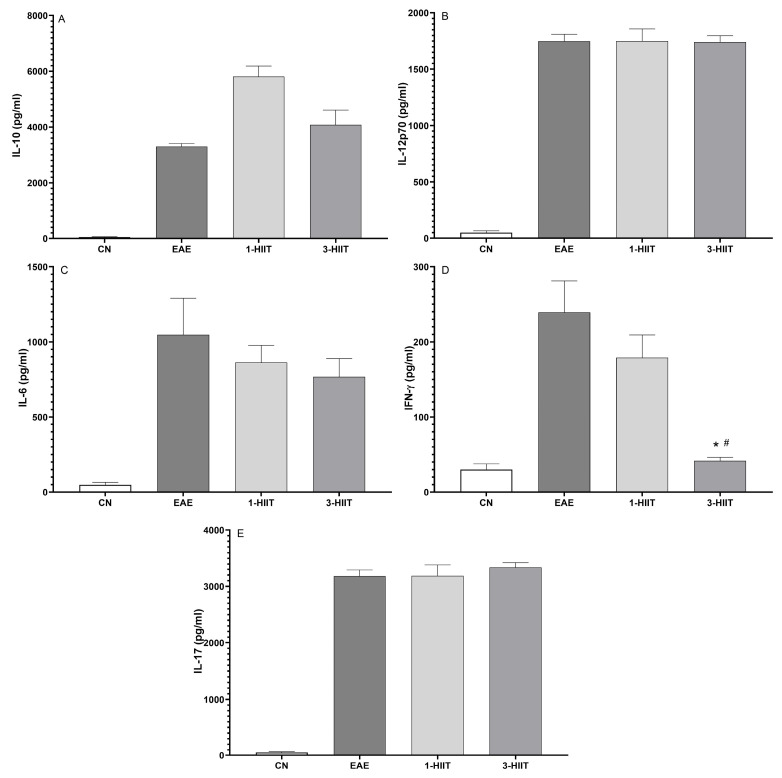
Cytokine levels in the supernatant of spinal cord homogenates from the experimental groups. (**A**) IL-10, (**B**) IL-12p70, (**C**) IL-6, (**D**) IFN-γ, and (**E**) IL-17. CN: Non-induced, non-exercised negative control; EAE: group induced with experimental autoimmune encephalomyelitis (EAE) and non-exercised; 1-HIIT: group induced with EAE and exercised using the 1-HIIT protocol; 3-HIIT: group induced with EAE and exercised using the 3-HIIT protocol. Number of animals per group: *n* = 8. * *p* < 0.05 versus EAE; # *p* < 0.05 1-HIIT versus 3-HIIT. Data are expressed as mean ± standard error of the mean (SEM). Significance assessed by one-way ANOVA with Fisher’s comparisons test or Kruskal–Wallis with Dunn’s comparisons test.

In the spinal cord (Figure 4A–E), the most significant difference was the reduced expression of IFN-γ in the 3-HIIT group compared with the other groups (Figure 4D).

### 2.4. Inflammatory Infiltrate in the Brain and Spinal Cord

**Figure 5:**
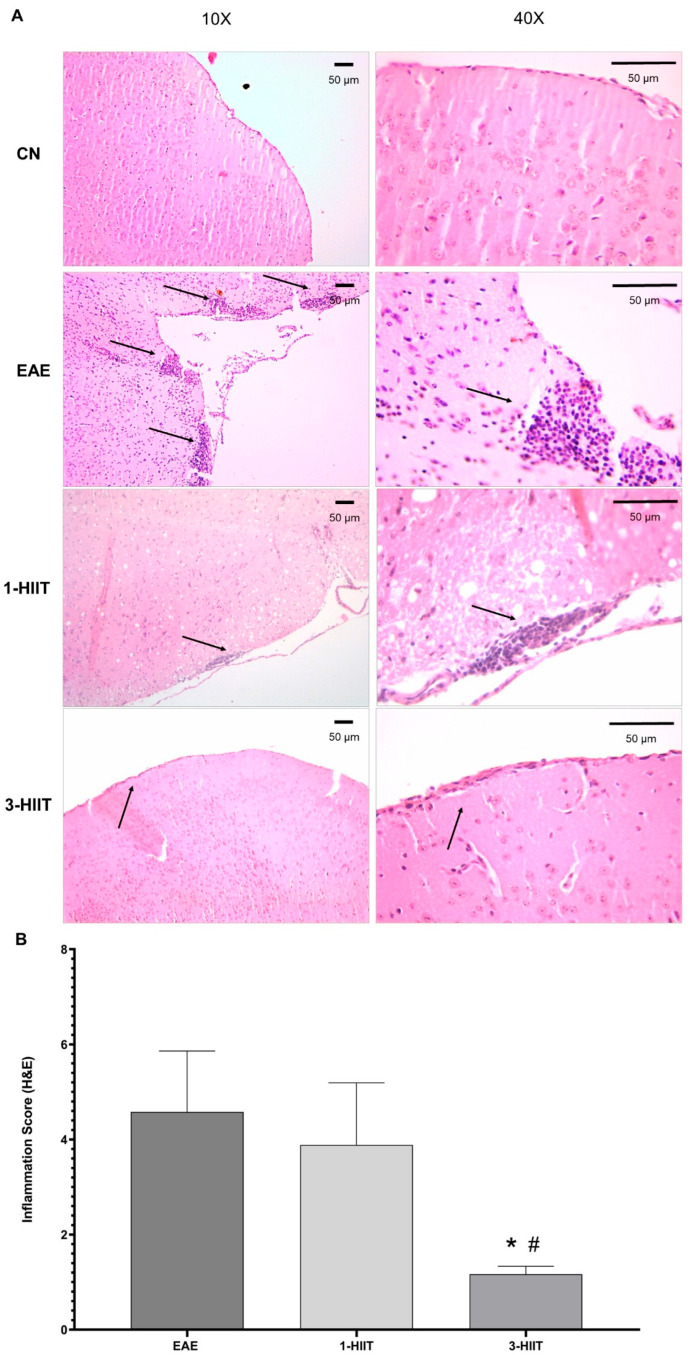
Histopathological analyses of the inflammatory process in brain sections stained with hematoxylin and eosin (H&E). (**A**) Brain sections are viewed at the objectives of 10× and 40× magnification, respectively. The arrows indicate the inflammatory foci. Bar = 50 µm scale bar. (**B**) Graph of the brain inflammation score, obtained by analyzing the sections from the different groups via blinded analysis. CN: Non-induced, non-exercised negative control; EAE: group induced with experimental autoimmune encephalomyelitis (EAE) and non-exercised; 1-HIIT: group induced with EAE and exercised using the 1-HIIT protocol; 3-HIIT: group induced with EAE and exercised using the 3-HIIT protocol. Number of animals per group: *n* = 4. * *p* < 0.05 versus EAE; # *p* < 0.05 1-HIIT versus 3-HIIT. Data are expressed as mean ± standard error of the mean (SEM). Significance assessed by one-way ANOVA with Fisher’s comparisons test or Kruskal–Wallis with Dunn’s comparisons test.

Histological analysis of brain sections reveals an intense inflammatory infiltrate in the EAE group (arrows) (Figure 5A); although to a lesser extent, an infiltrate is also present in the 1-HIIT group (Figure 5A). The inflammatory infiltrate is significantly reduced in the 3-HIIT group compared with both the EAE and 1 -HIIT groups (Figure 5A,B).

**Figure 6:**
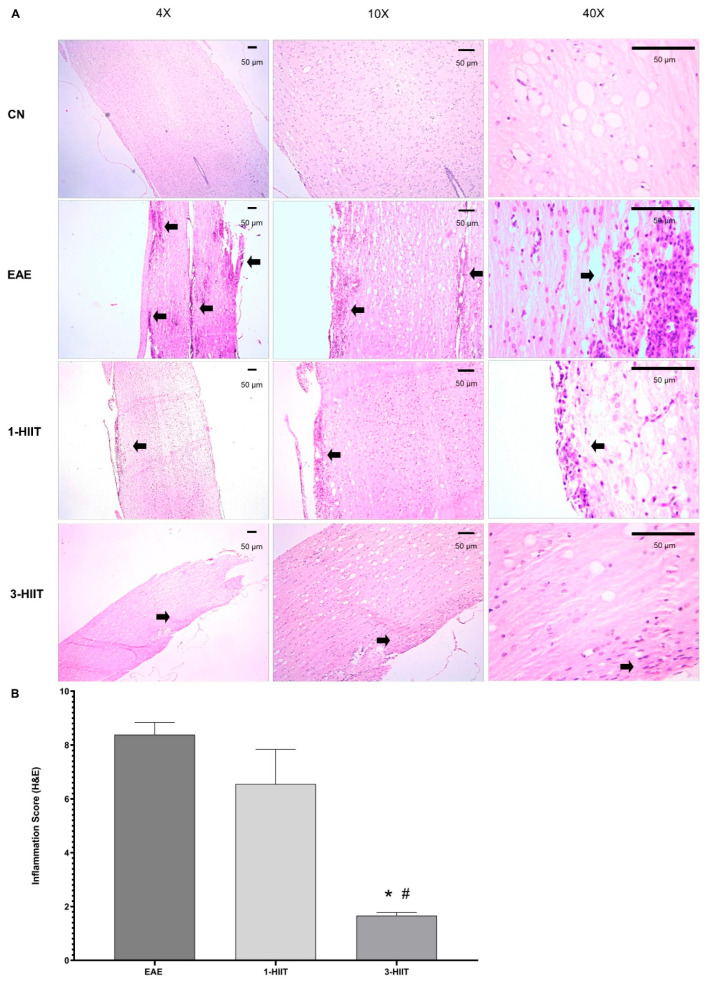
Histopathological analyses of the inflammatory process in longitudinal sections of the spinal cord using the hematoxylin and eosin (H&E) staining method. (**A**) Longitudinal sections of the spinal cord are viewed at the objectives of 4×, 10×, and 40× magnifications. The arrows indicate the inflammatory foci. Bar = 50 µm scale bar. (**B**) Graph of the inflammation score in longitudinal sections of the spinal cord, determined by blind analysis. CN: Non-induced, non-exercised negative control; EAE: group induced with experimental autoimmune encephalomyelitis (EAE) and non-exercised; 1-HIIT: group induced with EAE and exercised using the 1-HIIT protocol; 3-HIIT: group induced with EAE and exercised using the 3-HIIT protocol. Number of animals per group: *n* = 4. * *p* < 0.05 versus EAE; # *p* < 0.05 1-HIIT versus 3-HIIT. Data are expressed as mean ± standard error of the mean (SEM). Significance assessed by one-way ANOVA with Fisher’s comparisons test or Kruskal–Wallis with Dunn’s comparisons test.

Figure 6 shows longitudinal sections of the spinal cord stained using the H&E method. The images presented here mirror the pattern observed in the brain, with an inflammatory infiltrate present in the EAE and 1-HIIT groups, but significantly reduced in the 3-HIIT group (Figure 6A,B).

**Figure 7:**
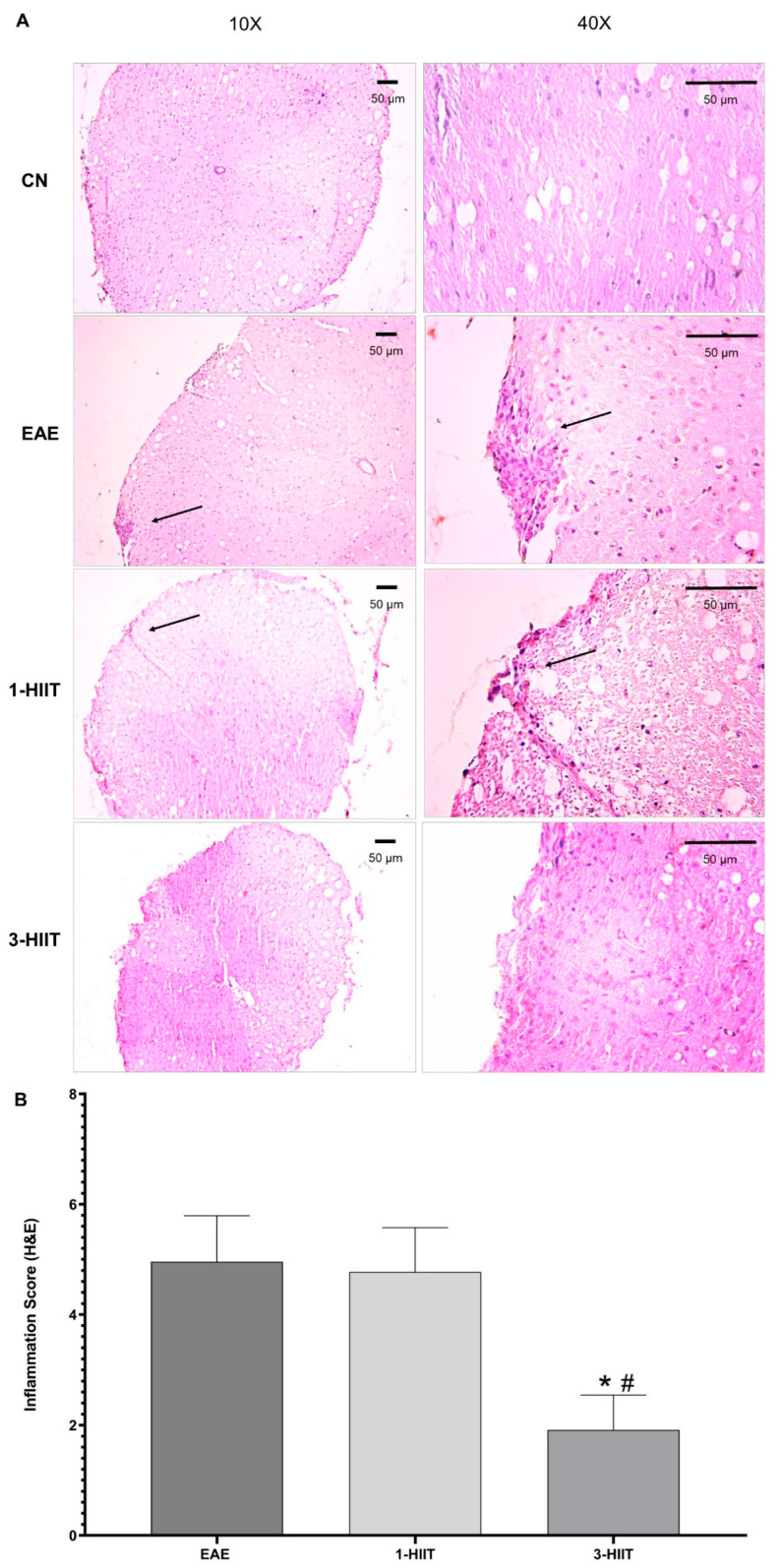
Histopathological analyses of the inflammatory process in cross-sections of the spinal cord using the HE method. (**A**) Cross-sections of the spinal cord are viewed at the objectives of 10× and 40× magnification, respectively. The arrows indicate the inflammatory foci. Bar = 50 µm scale bar. (**B**) Graph of the inflammation score for spinal cord cross-sections. CN: Non-induced, non-exercised negative control; EAE: group induced with experimental autoimmune encephalomyelitis (EAE) and non-exercised; 1-HIIT: group induced with EAE and exercised using the 1-HIIT protocol; 3-HIIT: group induced with EAE and exercised using the 3-HIIT protocol. Number of animals per group: *n* = 4. * *p* < 0.05 versus EAE; # *p* < 0.05 1-HIIT versus 3-HIIT. Data are expressed as mean ± standard error of the mean (SEM). Significance assessed by one-way ANOVA with Fisher’s comparisons test or Kruskal–Wallis with Dunn’s comparisons test.

Figure 7 shows cross-sections of the spinal cord stained using the H&E method. The images presented here reflect the pattern observed in the brain and in the cross-section of the spinal cord, with an inflammatory infiltrate present in the EAE and 1-HIIT groups, but significantly reduced in the 3-HIIT group (Figure 7A,B).

### 2.5. Spinal Cord Demyelination

**Figure 8:**
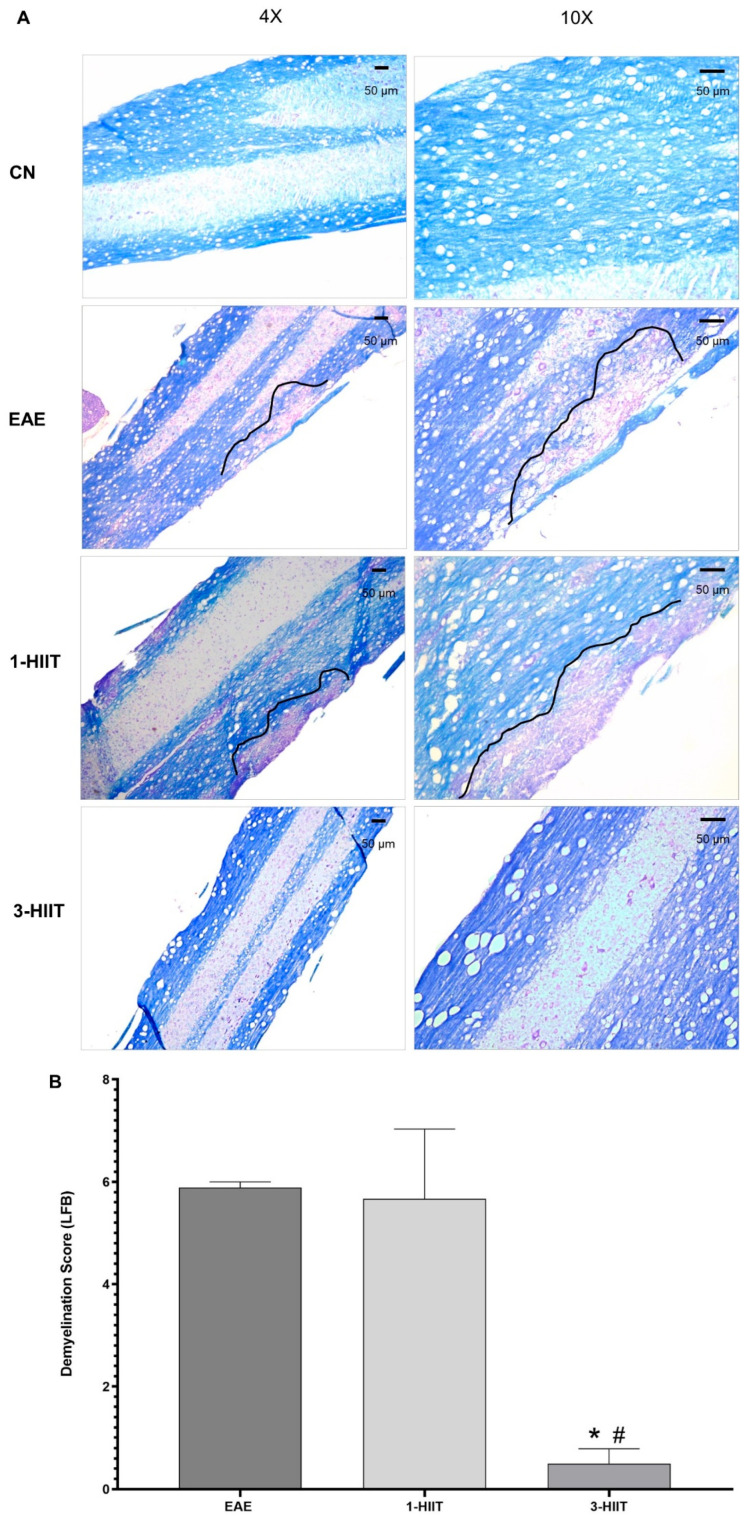
Histopathological analyses of the demyelination process in longitudinal sections of the spinal cord using the Luxol fast blue (LFB) method. (**A**) Spinal cord sections were viewed at the objectives of 4× and 10× magnification, respectively. The highlighted areas indicate demyelination processes. Bar = 50 µm scale bar. (**B**) Graph of the demyelination score in longitudinal sections of the spinal cord. CN: Non-induced, non-exercised negative control; EAE: group induced with experimental autoimmune encephalomyelitis (EAE) and non-exercised; 1-HIIT: group induced with EAE and exercised using the 1-HIIT protocol; 3-HIIT: group induced with EAE and exercised using the 3-HIIT protocol. Number of animals per group: *n* = 4. * *p* < 0.05 EAE versus 1-HIIT or versus 3-HIIT; # *p* < 0.05 1-HIIT versus 3-HIIT. Data are expressed as mean ± standard error of the mean (SEM). Significance assessed by one-way ANOVA with Fisher’s comparisons test or Kruskal–Wallis with Dunn’s comparisons test.

Histological analysis of spinal cord sections revealed demyelination in the EAE group (Figure 8A). Demyelination was less extensive in the 1 -HIIT group (Figure 8A) and was absent in the 3-HIIT group compared to both the EAE and 1-HIIT groups (Figure 8A,B).

**Figure 9:**
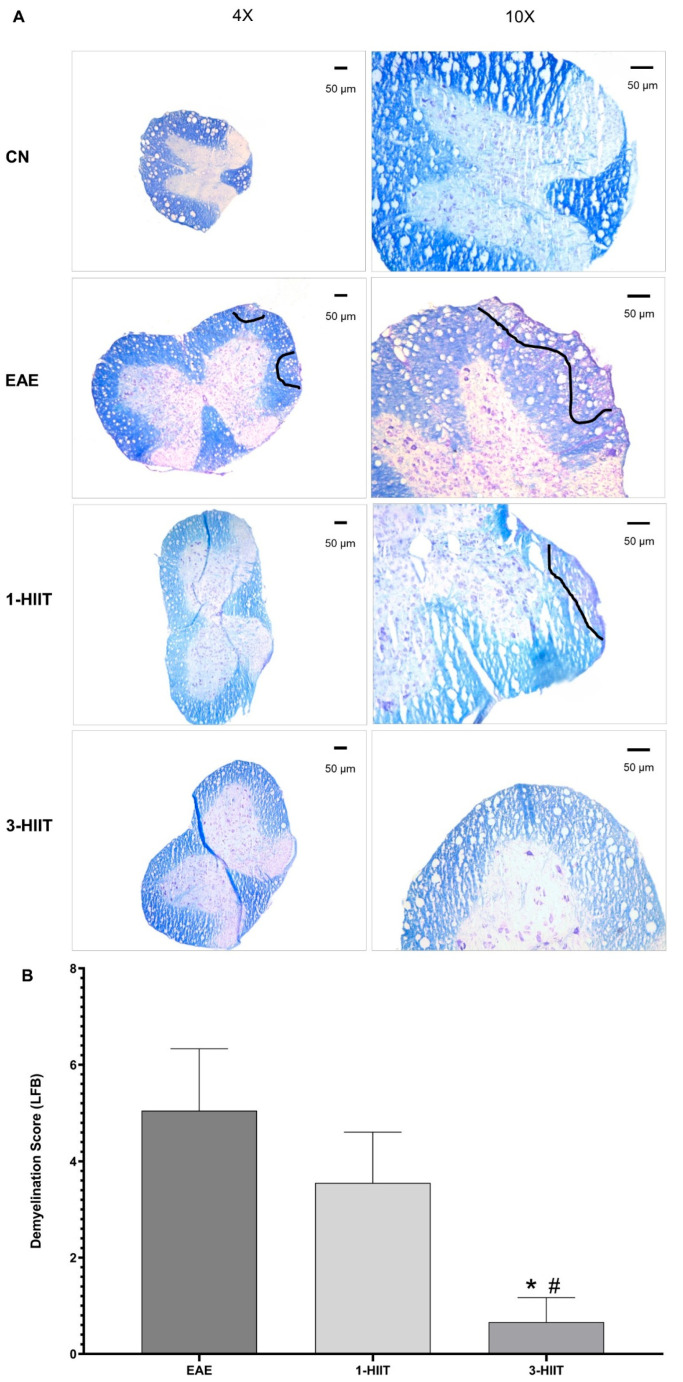
Histopathological analyses of the demyelination process in cross-sections of the spinal cord using the Luxol fast blue method. (**A**) Spinal cord sections were viewed at the objectives of 4× and 10× magnification, respectively. The highlighted areas indicate demyelination processes. Bar = 50 µm scale bar. (**B**) Graph of the demyelination score in cross-sections of the spinal cord. CN: Non-induced, non-exercised negative control; EAE: group induced with experimental autoimmune encephalomyelitis (EAE) and non-exercised; 1-HIIT: group induced with EAE and exercised using the 1-HIIT protocol; 3-HIIT: group induced with EAE and exercised using the 3-HIIT protocol. Number of animals per group: *n* = 4. * *p* < 0.05 versus EAE; # *p* < 0.05 1-HIIT versus 3-HIIT. Data are expressed as mean ± standard error of the mean (SEM). Significance assessed by one-way ANOVA with Fisher’s comparisons test or Kruskal–Wallis with Dunn’s comparisons test.

Figure 9 shows cross-sections of the spinal cord prepared using the LFB method, revealing demyelination in the EAE and 1-HIIT groups, but a significant reduction in the 3-HIIT group (Figure 9A,B).

## 3. Discussion

Physical exercise has been widely recognized as a strategy for preventing and managing chronic diseases [14]. In this context, HIIT has stood out for promoting significant improvements in cardiorespiratory fitness, glycemic control, blood pressure, and cardiac function compared with traditional forms of aerobic exercise [15]. As observed in the present study, previous results demonstrate that the 3 -HIIT protocol is superior to 1-HIIT for improving cardiovascular function [16].

In the context of EAE, a previous study demonstrated that mice subjected to HIIT exhibited an immunomodulatory response, resulting in attenuation of disease progression. This immunomodulation was associated with interference in the migration and invasion of lymph node-derived encephalitogenic cells (LNCs) by reducing Very Late Antigen-4 (VLA-4) via a systemic immune effect [18]. When comparing two distinct training models on neuromodulation in EAE: HIIT and HICT (high-intensity continuous training), a prior study found that both training models inhibited the encephalitogenic effect of autoreactive T cells. However, HICT reduced overall autoreactive T-cell proliferation, whereas HIIT specifically inhibited autoreactive Th1 and Th17 cells [19].

The results of the present study suggest that HIIT snacks (3 -HIIT) may have a more consistent effect on attenuating the inflammatory response in the central nervous system (CNS). When evaluating the CNS inflammatory infiltrate, the 3 -HIIT group had fewer inflammatory foci than the EAE and 1-HIIT groups. These findings are consistent with the clinical score and cytokine analyses, which showed increased IL-10 expression in the brain, associated with suppression of IL-12p70 in the brain and IFN-γ in the spinal cord. Interestingly, the 1-HIIT group exhibited significantly higher levels of IL-6 and IFN-γ in the brain, which initially appears paradoxical given the clinical improvement observed. This elevation may therefore reflect enhanced immunosurveillance rather than unchecked tissue damage. Despite having early onset, the 1-HIIT group showed lower clinical scores than the EAE group from 16 dpi onward. This could be explained by the context of ongoing subclinical autoimmunity—as occurs during the induction phase of EAE before visible clinical signs—this transient exercise-induced inflammatory milieu may have accelerated the migration of already-primed autoreactive lymphocytes across the blood–brain barrier, resulting in a slightly earlier clinical manifestation.

This cytokine profile may partly explain the reduced accumulation of inflammatory cells in the CNS and the low clinical score observed in the 3 -HIIT animals. In EAE, the presence of inflammatory infiltrates is directly related to disease activity and reflects the recruitment of LNCs into the CNS [19].

Regarding demyelination, the 3-HIIT group demonstrated even greater preservation of myelin sheath integrity, outperforming the EAE and 1-HIIT groups. This preservation could be explained by the activation of an anti-inflammatory profile that affects oligodendrocytes, favoring the survival of neural progenitor cell lines and their ability to regenerate the myelin sheath [7].

The fact that the 3-HIIT protocol performed better than the 1-HIIT protocol suggests that not only intensity but also the distribution of stimuli throughout the day may influence the biological response to exercise. Stimulus repetition may promote a more sustained immunoregulatory effect, contributing to more efficient control of inflammation in the CNS. This finding is consistent with previous studies demonstrating that more intense protocols tend to induce more robust immunoregulatory adaptations, which observed a reduction in demyelination, leukocyte infiltration, and inflammatory markers in the CNS associated with clinical improvement, reinforcing the potential of exercise as a strategy for modulating the immune response [18,19,20,21,22,23,24].

These findings open the door to considering physical exercise as a complementary strategy for managing multiple sclerosis, although further studies are needed to better understand the mechanisms involved and the feasibility in different disease courses. In addition, human studies indicate that HIIT is a safe and potentially effective strategy for improving functional and cardiorespiratory capacity in individuals with multiple sclerosis, especially those with a lower degree of disability [16]. A notable limitation of the present study is the exclusive use of female C57BL/6 mice. Although this choice was primarily motivated by the higher prevalence of multiple sclerosis in women (approximately 3:1 female-to-male ratio) and by the fact that all previously published studies on the effects of HIIT on EAE have been conducted in female animals [18,19,20], it precludes definitive conclusions regarding the potential influence of biological sex on the immunomodulatory outcomes of HIIT. In the MOG_35_–_55_/C57BL/6 EAE model specifically, ref. [25] reported no significant sex-related differences in the incidence of disease, clinical course, histological findings in the CNS, T cell response, and cytokine production of spleen cells to MOG, and anti-MOG IgG level in serum, suggesting that this particular model may be relatively sex-independent. Nevertheless, sexual dimorphism in CNS autoimmunity is well documented, with evidence of sex-specific genetic loci controlling EAE progression [26] and complex interactions between sex hormones, immune responses, and neuroinflammation [27].

Additionally, the present study did not monitor the animals’ estrous cycles, which represents another relevant limitation. The EAE model significantly disrupts estrous cycle homeostasis, providing experimental evidence for a direct causal link between neuroinflammation and reproductive hormonal perturbation. Given that the estrous cycle itself modulates EAE severity and that exercise training increases aromatase expression and local 17β-estradiol production, it is plausible that HIIT-induced immunomodulation may interact with the hormonal environment in a sex-dependent manner. Future studies from our group will include male C57BL/6 mice to determine whether the neuroprotective and immunoregulatory effects of accumulated HIIT, particularly the 3-HIIT protocol, exhibit sexual dimorphism and to elucidate potential interactions between sex hormones and exercise-induced immunomodulation in the context of autoimmune neuroinflammation.

Another important limitation of the present study is the absence of additional control groups, which would have enabled a more comprehensive interpretation of the results. Specifically, the study design did not include exercise-only control groups without EAE induction, which would have enabled disentangling exercise-induced effects from their interactions with autoimmune neuroinflammation. Without these groups, we cannot definitively determine whether the observed cytokine changes in trained EAE animals reflect an interaction between exercise and the disease state or an independent effect of physical activity on the immune system. Furthermore, the absence of an adjuvant-only control group (CFA + pertussis toxin without MOG) is a significant limitation, as CFA alone has been shown to induce glial activation, neuroinflammation, and pain responses, thereby confounding EAE studies. The lack of this control prevents us from ruling out that some of the observed inflammatory changes may be attributable to the adjuvant rather than to MOG-induced autoimmunity. Future studies should incorporate control groups to dissect the independent contributions of exercise, adjuvant-induced inflammation, and the autoimmune pathology itself, thereby providing a more complete mechanistic understanding of HIIT’s effects on neuroinflammatory diseases.

## 4. Materials and Methods

### 4.1. Animals

For the experiment, 48 female C57BL/6 conventional mice, aged 5 to 6 weeks, were used. The mice were obtained from the Central Animal Facility of the Federal University of Viçosa (UFV). The animals were housed in the animal facility of the School of Medicine at the Federal University of the Jequitinhonha and Mucuri Valleys (UFVJM) in Teófilo Otoni, Minas Gerais. They were housed in ventilated cages, with food and water provided ad libitum, under a 12 h light/dark cycle. To stimulate natural digging and shelter-seeking behavior, environmental enrichment was provided. All experimental procedures followed the principles established by the Brazilian Code for the Use of Animals in Research, in accordance with the ARRIVE guidelines, and were previously approved by the Animal Ethics Committee (CEUA) of Mucuri/UFVJM under Protocol No. 01/2023R on 19 July 2023.

### 4.2. Training Protocol

**Figure 10:**
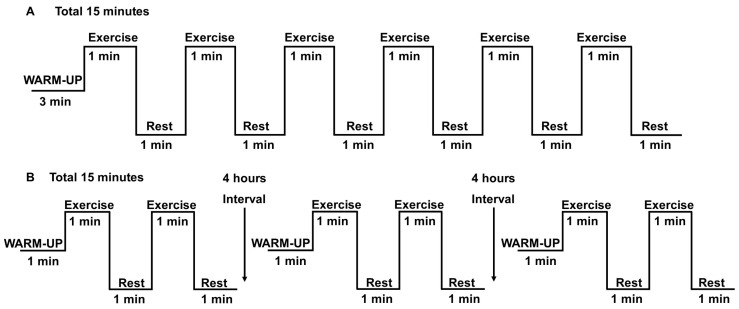
Schematic representation of the HIIT protocols used. (**A**) Group 1-HIIT. (**B**) Group 3-HIIT. Adapted from De Sousa, 2022 [28].

Female C57BL/6 mice were randomly assigned to four experimental groups, each containing 12 animals: Group 1 (CN): Negative Control. The animals in this group were not induced with EAE and did not perform physical exercise. Group 2 (EAE): EAE Control. The animals were induced with EAE but did not engage in physical exercise. Group 3 (1-HIIT): EAE with High-Intensity Interval Training (HIIT) in a single daily session. The animals were induced with EAE and underwent one HIIT session per day (15 min). Group 4 (3-HIIT): EAE with HIIT snacks, in three shorter daily sessions. The groups were divided before the training began. The allocation was based on the final mean weight of each group, with approximately equal mean weights across groups. The group size was calculated based on previous studies [28,29]. The animals were induced with EAE and underwent three daily HIIT sessions (5 min each) with a 4 h interval. After 7 days of acclimatization and adaptation to the animal facility, the mice underwent treadmill (Insight Equipamentos, Ribeirão Preto, Brazil) habituation for the experiment. Subsequently, the training volume for each animal was determined using a maximum-load test. The maximum-load test involved exposing the animals to exercise, with the speed (m/min) increased by 3 units every 2 min. During this process, the animals’ level of exhaustion was assessed according to the parameters established in the protocol, thereby ending the exercise and defining each animal’s individual maximum speed and, subsequently, the training group’s average. A new maximum load test was required after the 4th week of training, by which time these animals would likely have achieved better physical conditioning. The total training period was 9 weeks over 5 consecutive days (Monday to Friday). Consequently, each 1-HIIT subgroup (*n* = 4) performed separate daily sessions at different times (at 8 a.m., 12 p.m., and 4 p.m.), with a 4 h interval between each session to eliminate any circadian influences. Each training session consisted of a 3 min warm-up at 50% of Vmax, followed by 6 sets of 1 min exercise at 85% of Vmax, alternating with 1 min rest periods (passive recovery), for a total of 15 min of training. The 3-HIIT group had its training split into shorter sessions (5 min each), consisting of a 1 min warm-up, two sets of exercises at 85% of Vmax, with 1 min of passive recovery between each set. Although performing shorter sessions, this group trained 3 times a day, also totaling 15 min of daily HIIT. Training progression occurred through weekly increases in the intensity of the HIIT sessions, but without altering the training volume, according to the following schedule: Week 1—6 × 1 min at 85% Vmax; Week 2—90% Vmax; Week 3—95% Vmax; Week 4—100% Vmax. The same training protocol was established following the second maximum-load test (Figure 10).

### 4.3. EAE Induction

After 6 weeks of training, the mice were induced to develop EAE by administering 100 μL of a solution containing 100 μg of oligodendrocyte myelin glycoprotein (MOG35-55—GENONE—Rio de Janeiro, Brazil) diluted *v*/*v* with Complete Freund’s Adjuvant (CFA—Santa Cruz Biotechnology, Dallas, TX, USA) containing 400 μg of Mycobacterium tuberculosis (H37RA—BD, Franklin Lakes, NJ, USA) at 4 mg/mL. In addition, the animals also received an intraperitoneal injection of 100 μL of pertussis toxin (300 ng—Thermo Fisher Scientific, Waltham, MA, USA), which was administered again after 48 h.

### 4.4. Clinical Score, Body Weight, and Food Consumption Evaluation

The score used to assess disease progression in mice was based on four main parameters related to neurological status, with signs of impairment assessed in a caudocranial direction. The score was the sum of the parameters assessed (Table 1). The score was assessed daily, starting on the 9th day after EAE induction until day 21st. Dead animals were excluded from the experiment, and their data were not analyzed. In addition, body weight was measured every 2 days from the start of training, and food intake was assessed weekly. At day 21st, euthanasia was performed using a lethal dose of xylazine (10 mg/kg—Syntec, Tamboré, Brazil) and ketamine (100 mg/kg—Syntec) administered intraperitoneally. 4 animals/group underwent cardiac perfusion with a 4% paraformaldehyde solution to harvest the brain and spinal cord for histological analysis. 8 animals/group were perfused with cold phosphate-buffered saline (PBS) to harvest the brain and spinal cord for cytokine quantification by ELISA. The harvested tissues were rapidly stored in a freezer at −80 °C.

### 4.5. Cytokine Quantification (ELISA)

After the animals were euthanized, the collected tissues were weighed and macerated with 0.4 M sodium chloride (NaCl—Isofar Indústria e Comércio, Duque de Caxias, Brazil), 0.05% Tween 20 (Isofar Indústria e Comércio), 0.5% bovine serum albumin (Merck Group, Darmstadt, Germany), 0.1 M phenylmethylsulfonyl fluoride (Merck Group), 0.1 M benzethonium chloride (Merck Group), 10 mM ethylenediaminetetraacetic acid (EDTA—Merck Group), and 20 pM aprotinin (Merck Group). The mixture was centrifuged, and the supernatant was collected. Subsequently, the cytokines IL-6, IL-10, IL-12, IL-17, and IFN-γ in brain and spinal cord supernatants were quantified using the ELISA method according to the protocol provided with the BD OptEIA Kit (BD). The plates were read in a microplate reader at 450 nm (EZReady 2000, Biochrom, Holliston, MA, USA).

### 4.6. Histological Analyses

The presence and extent of the inflammatory infiltrate in the collected tissues were assessed using H&E (hematoxylin and eosin—Merck Group) staining of spinal cord (transversal and longitudinal cuts) and brain samples (transversal cuts), which were also analyzed for demyelination using the Luxol Fast Blue (LFB) method. The specimens were fixed in 4% paraformaldehyde (Merck Group), embedded in paraffin (Merck Group), and sectioned at 10 μm. The sections were stained with LFB, differentiated with lithium carbonate (Merck Group) and 70% ethanol (Merck Group), and counterstained with cresyl violet (Merck Group). The slides were analyzed using an Olympus BX51 microscope (Olympus Corporation, Tokio, Japan), with images acquired with Image-Pro Plus (version 6.0, Media Cybernetics, Rockville, MD, USA). Both staining were evaluated semi-quantitatively and in a blinded manner, using a scale of 0 to 3, 0 = no demyelination/inflammation (no sites of demyelination/inflammation observed in the analyzed field); 1 = focal demyelination/inflammation (a single isolated site of demyelination/inflammation observed in the analyzed field); 2 = multifocal demyelination/inflammation (more than one site of demyelination/inflammation observed in the analyzed field); 3 = extensive demyelination/inflammation (multiple sites of demyelination/inflammation observed in the analyzed field), adapted from [31]. Three slides from each organ were selected. On each slide, four quadrants were analyzed and scored on a scale of 0 to 3. The slide score was the sum of the quadrants evaluated. The animal score was the mean of the three slides. The result score was the mean ± standard error of the mean (SEM) of the animals in the group.

### 4.7. Statistical Analyses

Statistical analysis was conducted based on the presentation of mean results accompanied by standard error. Data distributions were evaluated using the Kolmogorov–Smirnov and Shapiro–Wilk tests. Comparisons between experimental groups were performed using parametric or nonparametric tests, including one-way analysis of variance (ANOVA), two-way ANOVA, and the Kruskal–Wallis test, depending on the data’s distribution. In all analyses, *p* < 0.05 was considered statistically significant. Statistical analysis of the data was performed using GraphPad Prism software, version 5.0 (GraphPad Software Inc., Boston, MA, USA).

## 5. Conclusions

Therefore, the data obtained in this study provide consistent evidence supporting the interpretation that HIIT snacks can promote a significant reprogramming of the autoimmune response in the CNS of mice. This enhancement was likely due to modulation of inflammatory mediators and control of cellular infiltration into nervous tissue. Thus, the findings presented not only reinforce the therapeutic potential of physical exercise for modulating neuroinflammation but also broaden the prospects for future research. It is therefore important to conduct more in-depth studies that elucidate the molecular and cellular mechanisms underlying this process, contributing to a comprehensive understanding of the effects of HIIT on CNS autoimmune diseases.

## Figures and Tables

**Table 1:** Clinical neurological scale for assessing EAE. Adapted from: De Paula et al., 2008 [30].

Score ^1^	Clinical Sign	Body Part
0	No clinical sign	Tail
1	Loss of tail tonus	
2	Paralysis	
1	Weakness of one paw	Posterior limbs
2	Weakness of both paws	
3	Paralysis of one paw	
4	Paralysis of both paws	
0	No clinical sign	Anterior limbs
1	Weakness of one or both paws	
2	Paralysis of one or both paws	
0	Continence	Bladder
1	Incontinence	

^1^ The final score was the sum of the parameters assessed.

## Data Availability

The raw data supporting the conclusions of this article will be made available by the authors on request.

## Abbreviations

The following abbreviations are used in this manuscript:

HIIT	high-intensity interval training
EAE	experimental autoimmune encephalomyelitis (EAE)
CN	Negative control
CNS	Central Nervous System
MS	Multiple Sclerosis
MOG	myelin oligodendrocyte glycoprotein
DPI	Days post-induction
IL	Interleukin
IFN-γ	Interferon gamma
H&E	Hematoxylin and eosin
LFB	Luxol fast blue
CFA	Complete Freund’s Adjuvant
